# Induction of superficial zone protein (SZP)/lubricin/PRG 4 in muscle-derived mesenchymal stem/progenitor cells by transforming growth factor-β1 and bone morphogenetic protein-7

**DOI:** 10.1186/ar3793

**Published:** 2012-04-10

**Authors:** José A Andrades, Shirley C Motaung, Pedro Jiménez-Palomo, Silvia Claros, José M López-Puerta, José Becerra, Thomas M Schmid, A Hari Reddi

**Affiliations:** 1 Department of Cell Biology, Genetic and Physiology, Faculty of Sciences, Networking Biomedical Research Center in Bioengineering, Biomaterials and Nanomedicine (CIBER-BBN), University of Málaga, 29071-Málaga, Spain; 2 Department of Biomedical Sciences, Tshwane University of Technology, Faculty of Sciences, Private Bag X680, Pretoria 0001, South Africa; 3 Department of Orthopaedic Surgery and Traumatology, Universitary Hospital Virgen del Rocío, 41013-Sevilla, Spain; 4 Department of Biochemistry, Rush University Medical Center, Chicago, IL 60612, USA; 5 Lawrence Ellison Center for Tissue Regeneration and Repair, Department of Orthopaedic Surgery, University of California, Davis, Sacramento, CA 95817, USA

## Abstract

**Introduction:**

Articular cartilage (AC) is an avascular tissue with precise polarity and organization. The three distinct zones are: surface, middle and deep. The production and accumulation of the superficial zone protein (SZP), also known as lubricin, by the surface zone is a characteristic feature of AC. To date, there is a wealth of evidence showing differentiation of AC from mesenchymal stem cells. Most studies that described chondrogenic differentiation did not focus on AC with characteristic surface marker SZP/lubricin. The present investigation was initiated to determine the induction of SZP/lubricin in skeletal muscle-derived mesenchymal stem/progenitor cells (MDMSCs) by transforming growth factor-β1 (TGF-β1) and bone morphogenetic protein-7 (BMP-7).

**Methods:**

MDMSCs were cultured as a monolayer at a density of 1 × 10^5 ^cells/well in 12-well tissue culture plates. Cell cultures were treated for 3, 7 and 10 days with TGF-β1 and BMP-7. The medium was analyzed for SZP. The cells were used to isolate RNA for RT-PCR assays for SZP expression.

**Results:**

The SZP/lubricin increased in a time-dependent manner on Days 3, 7 and 10 in the medium. As early as Day 3, there was a three-fold increase in response to 3 ng/ml of TGF-β1 and 300 ng/ml of BMP-7. This was confirmed by immunochemical localization of SZP as early as Day 3 after treatment with TGF-β1. The expression of SZP mRNA was enhanced by TGF-β1.

**Conclusions:**

The present investigation demonstrated the efficient and reproducible induction of SZP/lubricin accumulation by TGF-β1 and BMP-7 in skeletal MDMSCs. Optimization of the experimental conditions may permit the utility of MDMSCs in generating surface zone-like cells with phenotypic markers of AC and, therefore, constitute a promising cell source for tissue engineering approaches of superficial zone cartilage.

## Introduction

Articular cartilage (AC) is an avascular tissue with feeble regeneration potential, possibly because of the lack of vasculature, the innate mechanisms of tissue repair by humoral factors, and recruitment of stem/progenitor cells does not occur [1]. Osteoarthritis (OA) is a common degenerative joint disease affecting the AC in aged individuals. Currently there are limited treatment options, other than total joint replacement. However, smaller injuries to AC in younger patients can be treated with cell-based therapies, such as autologous chondrocyte implantation (ACI) or bone marrow-derived mesenchymal stem cell (MSC) transplantation [2,3].

Muscle-derived mesenchymal stem cells (MDMSCs) are a potentially useful source of cells for the induction of synthesis and secretion of superficial zone protein (SZP), a characteristic marker of the surface zone of the AC. In view of the location, accessibility and great expandability, MDMSCs are a promising cell source for creating a superficial zone of AC by tissue engineering [4]. We have hypothesided that MDMSCs could be induced by TGF-β1 and BMP 7 to differentiate into superficial zone cartilage-like cells that synthesize SZP/lubricin/PRG 4. TGF-β1 increased SZP accumulation in the media in a dose-dependent manner. The increased SZP accumulation was also reflected by increased SZP mRNA expression as assessed by RT-PCR. The increased accumulation of SZP was confirmed by immunochemical localization. SZP protein accumulation was stimulated by BMP-7 in a dose-dependent manner. In this investigation we demonstrate that the MDMSCs can be induced to secrete SZP into the medium.

## Materials and methods

### Cell culture

Skeletal muscle was obtained from the hind limbs of four female four-week-old Wistar rats (Charles River France, L'arbresle Cedex France). The surrounding soft tissue was dissected and the samples were washed, enzymatically digested and the cells cultured following a conventional protocol. The medium was changed two times/week and the cells selected by their capacity to attach to the dish surface, discarding the floating cells at the first medium change at 72 h [5]. The surgical procedure was approved by the Ethics Committee of the University of Málaga, Spain.

### Flow cytometry analysis of cells

In order to analyze and confirm the expression of surface markers characteristic for MSCs on rat MDMSCs, flow cytometry analysis using specific fluorochrome-conjugated monoclonal antibodies was used. Adherent cells at passage 1 were washed in PBS, harvested in trypsin/EDTA and in flow cytometry (fluorescence-activated cell sorting, FACS) buffer. Cells aliquots (1 × 10^6 ^cells) were incubated in FACS buffer containing monoclonal antibodies to phycoerythrin (PE)-conjugated CD29 (integrin alpha-1 involves in cell adhesion mechanism), fluorescein isothiocyanate (FITC)-conjugated CD34, allophycocyanine (APC)-conjugated CD45 (both specific for hematopoietic cells) and alkaline phosphatase (ALP, all from R&D Systems, Minneapolis, MN, USA), FITC-conjugated CD166 (AbD Serotec. Specific antigen was used for MSCs) and STRO-1 (R&D Systems' characteristic antigen for MSCs) with a PE-conjugated antimouse IgM (AbD Serotec), or an appropriate isotype control antibody (Sigma, St. Louis, MO, USA). After 30 minutes in the dark on ice, cells were washed again in FACS buffer before flow cytometry analysis. A total of 400,000 events/sample were analyzed on a *MoFlo^® ^*SP1338 (DakoCytomation, Glostrup, Denmark) using Summit software. Cells were gated on forwards and side scatter to exclude debris and cell aggregates, and dead cells were excluded by 7-Amino-Actinomycin D (7-AAD. BD Pharmigen, Franklin Lakes, NJ, USA) staining.

### Monolayer culture

The MDMSCs were plated as monolayers at a density of 1 × 10^5 ^cells/well in 12-well culture plates (Corning, Lowell, MA, USA). Cells were cultured in media with 10% serum overnight to permit cell attachment. The next day media was changed to serum-free medium and cells were treated with various morphogens according to the experimental design.

### Experimental design

Monolayer cell cultures were treated for 3, 7 and 10 days with the following growth factors and morphogens: TGF-β1 (R&D Systems, Minneapolis, MN, USA) at 1 ng/ml, 3 ng/ml and 10 ng/ml), and BMP-7 (a generous gift from Dr. D. Rueger, Stryker Biotech, Hopkinton, MA, USA), at 100 ng/ml, 300 ng/ml and 1,000 ng/ml). At Days 7 and 10 group half of the culture medium was substituted on Day 3 and replaced with fresh medium supplemented with the same concentration of the different morphogens. This method ensured the preservation of the conditioned medium with secreted autocrine factors at every medium change. The medium were collected on Days 3, 7 and 10 for SZP analyses by ELISA. The cells were used to isolate RNA for RT-PCR. The concentrations of morphogens were based on previous studies [6].

### Real-time reverse transcription-polymerase chain reaction

Total RNA was extracted from cell culture using an RNeasy mini kit (Qiagen, Valencia, CA, USA) with on-membrane DNase 1 (Qiagen) digestion to avoid genomic DNA contamination. Total RNA was reverse transcribed into single-strand cDNA using Superscript First-Strand Synthesis System with Oligo (dT) _primers _(Invitrogen, Carlsbad, CA, USA). Real-time quantitative PCR reactions were performed in triplicate on cDNA with an ABI Prism 7700 Sequence Detection System and PCR Taqman Universal Mastermix reagents (Perkin Elmer/Applied Biosystems, Foster City, CA, USA) following the recommended protocols. Superficial zone protein (SZP) mRNA levels were normalized to glyceraldehydes 3-phosphate dehydrogenase (GAPDH) levels and expressed relative to the control (untreated) culture levels (ΔΔ*C_T _*methods, Applied Biosystems). The primers for SZP (Rn01490812_ml) and GAPDH (4308313). were designed by Applied Biosystems.

### Enzyme-linked immunosorbent assay (ELISA): analysis of SZP protein level

SZP accumulation in the culture medium was quantified by sandwich ELISA using purified SZP as standard. Each well of 96-well MaxiSorp plates (Nalge Nunc International, Rochester, NY, USA) was coated with 1 μg/ml peanut lectin (EY Labo Laboratories, San Mateo, CA, USA) in 50 m*M *sodium carbonate buffer (pH 9.5) [7]. Then, the wells were blocked with 1% bovine serum albumin in the same buffer. Aliquots of culture medium were incubated in the wells. The wells were incubated with 2 μg/ml of monoclonal antibody S6.79 and next with horseradish peroxidase-conjugated goat anti-mouse IgG. Wells were washed with phosphate buffered saline containing 0.05% Tween 20 (Sigma) between all steps. Finally, the TMB Peroxidase EIA Substrate Kit (Bio-Rad, Hercules, CA, USA) was used and the absorbance of each well was measured. SZP levels were calculated using an SZP standard, which was purified by affinity chromatography on a peanut lectin column; purity was verified by Immunoblot analysis and quantified using a Micro BCA Protein Assay Kit (Pierce Biiotechnology, Rockford, IL, USA).

### Immunolocalization of SZP

Freshly isolated culture cells were fixed with 4% paraformaldehyde (Fisher Scientific, Pittsburgh, PA, USA), and peroxidase was blocked with 3% hydrogen peroxide. The cultures were probed for SZP using monoclonal antibody (mAb) S6.79 (a generous gift from Dr. T. Schmid, Rush Medical College, Chicago, IL, USA) with a 1:250 dilution then incubated overnight at 4°C. Thereafter, cells were incubated with biotinylated secondary antibodies (Horseradish peroxidase-conjugated goat anti-mouse IgG (1:100) dilution. In control experiments, no primary antibody was applied. Standard immunohistochemical staining was performed using Vecatatin^® ^ABC reagent and ImmPACT™ diaminobenzine peroxidase substrate (Vector Laboratories, Burlingame, CA, USA) resulting in a brown precipitate. No counterstaining was performed.

### Statistical analysis

All results are presented as averages and standard deviations for each experiment (*n *= 6). A one-way analysis of variance (ANOVA) with Tukey's HSD (Honestly Significant Differences) to account for multiple comparisons was used to determine the effects of TGF-β1 and BMP-7. A significance level of *P *< 0.05 was used to determine the difference between the control and treated cells and the dose-dependence of growth factors.

## Results

### Characterization of MDMSCs

**Figure 1:**
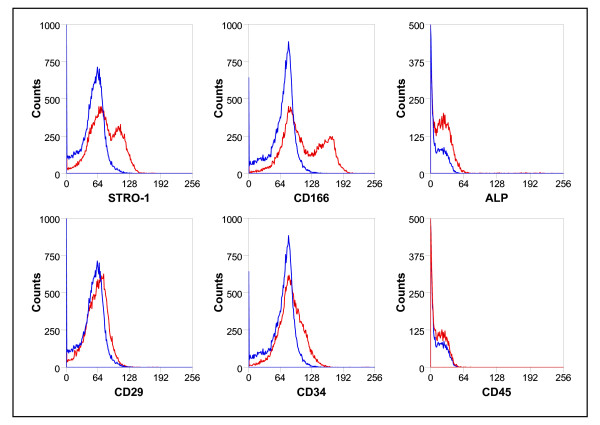
**Phenotype of the cultured rat muscle-derived mesenchymal stem/progenitor cells (MDMSCs)**. The MDMSCs were STRO-1 and CD166 positives, and negatives for Alkaline Phosphatase (ALP), CD29, CD34, and CD45. The blue line represents the corresponding control isotype.

The cell populations from MDMSCs were characterized by staining for surface marker proteins. As shown in Figure 1, we have found a relative heterogeneous cell population since the majority of the cells stained positive for STRO-1 and CD166, and negative for CD29, CD34 and CD45, while some cells were positive for ALP.

### SZP expression by MDMSCs

**Figure 2:**
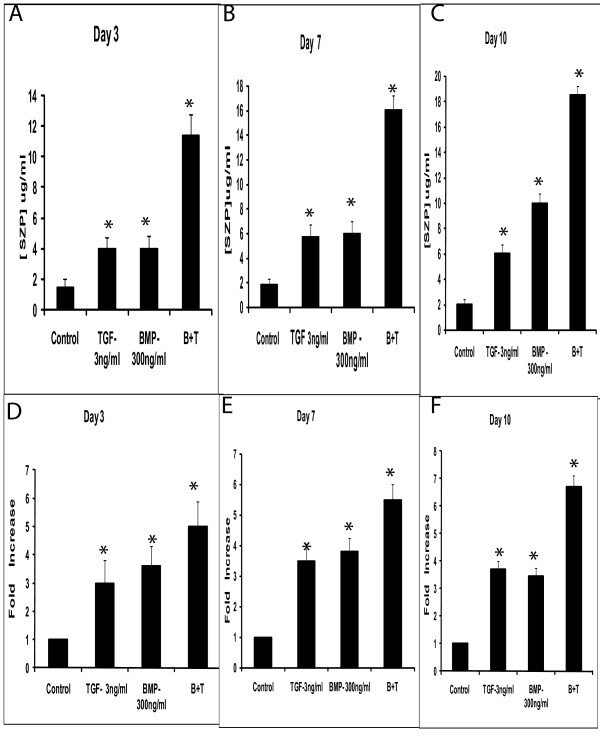
**Effects of TGF-β1 and BMP-7 on SZP in the muscle-derived mesenchymal progenitor/stem cells (MDMSCs)**. Primary cells were cultured for 3 days (A and D), 7 days (B and E) and for 10 days (C and F) as a monolayer in serum-free conditioned medium with TGF B1 (3 ng/ml) and BMP-7 (300 ng/ml). SZP in the medium was quantified by enzyme-linked immunosorbent assay (ELISA) and SZP mRNA induction was assessed using quantitative real-time reverse transcriptase polymerase chain reaction (RT-PCR). Values were represented by the means ± standard deviations. A significance level of *P *< 0.05 was used to determine the differences between the control and treated cells.

The accumulation of SZP in the culture medium increased in a time-dependent manner as in confirmation of earlier studies [7]. The culture media were analyzed on Days 3, 7 and 10. ELISA analysis revealed that MDMSCs synthesized and secreted SZP into the culture medium (Figure 2A-C). TGF-β1 exhibited a robust capacity to stimulate SZP accumulation. BMP-7 also significantly up-regulated the accumulation of SZP. On Day 3, compared to the control, there was a three-fold increase by treatment of 3 ng/ml TGF-β1, and similar response to 300 ng/ml BMP-7. There was a synergistic action of TGF-β1 and BMP-7 for accumulation of SZP. Similar results were observed at Days 7 and 10 (Figure 2B, C).

The expression of SZP mRNA in MDMSCs was enhanced by concurrent treatment of 3 ng/ml TGF-β1 and 300 ng/ml BMP-7 during the entire experiment period, on Day 10 (Figure 2C, F). Stimulation of SZP expression reached the maximal level at Day 10 with a four-fold increase over control. Treatment with TGF-β1 alone enhanced the expression of SZP mRNA during the entire experimental period. The expression of SZP mRNA and protein level was higher throughout the experiment in response to BMP-7 on Days 3, 7 and 10.

**Figure 3:**
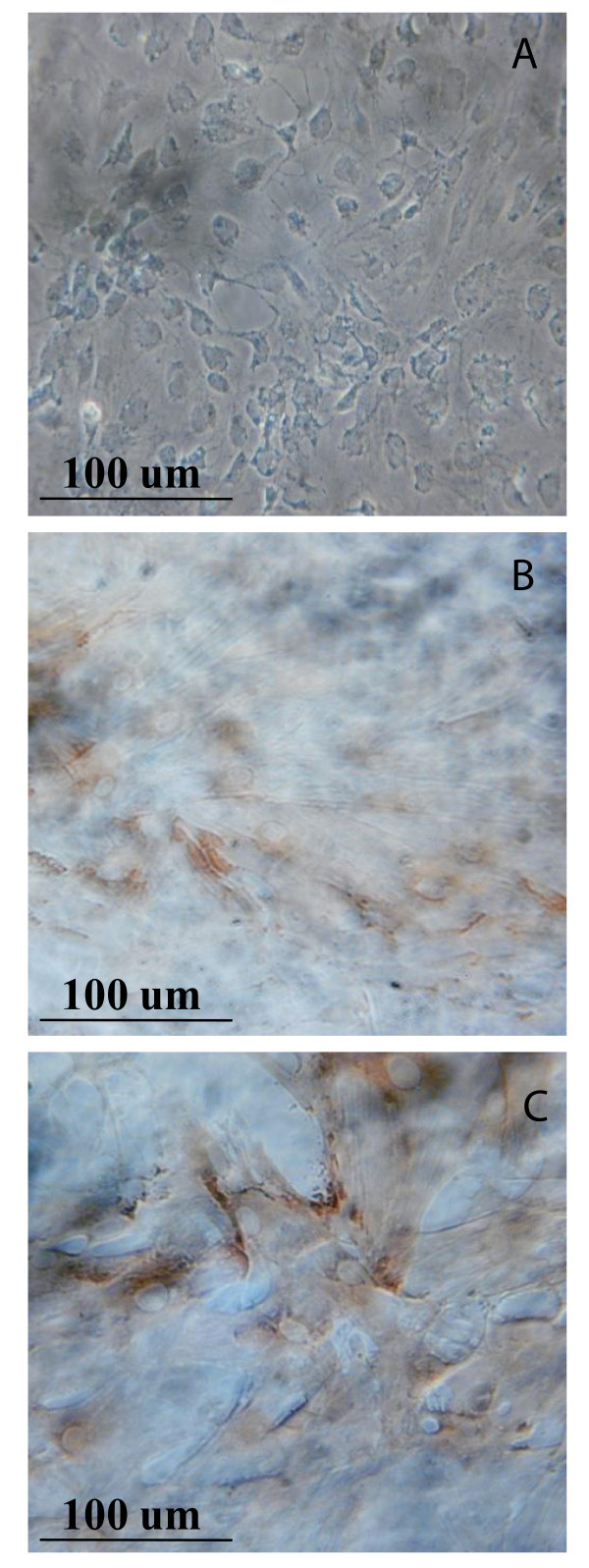
**Immunolocalization of the SZP in the rat muscle-derived mesenchymal stem/progenitor cells (MDMSCs)**. Control (**A**), 3 ng/ml of TGF-β1 (**B**), and 300 ng/ml BMP-7 (**C**), treated for three days.

MDMSCs cultures in monolayers were examined by immunocytochemistry for SZP using a monoclonal antibody. Treatment of cells with both morphogens TGF-β1 and BMP 7 at the referred concentrations stimulated the expression of SZP as early as Day 3 (Figure 3).

## Discussion

AC is an anisotropic structure and the surface, middle and deep zones are heterogeneous in organization. The chondrocyte phenotype, cell shape and the extracellular matrix structure varies between the different zones of this anisotropic tissue. The surface zone chondrocytes secrete SZP, also known as lubricin or PRG 4, and plays a key role in lubrication.

The present investigation demonstrated that SZP is synthesized and secreted by MDMSCs, indicating the potential utility of stem cells in restoring functional lubrication. We investigated the induction of SZP expression in primary MDMSCs by using TGF-β1 and BMP-7 in a monolayer culture system. BMP-7 stimulated SZP accumulation, although the effect was modest compared to TGF-β1. Concurrent treatment with TGF-β1 and BMP-7 resulted in significantly higher expression of SZP compared with the control group. These trends in regulation of SZP expression were similar to those previously observed in bovine synovial cells [7], mesenchymal progenitors in the infrapatellar fat pad of the knee joint [8], and in human MSCs, either by their differentiation into chondrocytes through commercial chondrogenic medium [9], or by transduction with an adenoviral vector [10]. The stimulation of SZP secretion in MDMSCs by TGF-β1 and/or BMP-7 demonstrates an efficient cell source that could contribute to regeneration of damaged AC and ameliorate the pathology of joint function in OA.

Another remarkable finding of this study is that expanded MDMSCs had the ability to synthesize and secrete SZP after *in vitro *differentiation. The ELISA analysis showed the presence and up-regulation of the SZP accumulation in media at 10 days of induction. Equally, real-time PCR analysis showed significant up-regulation of SZP mRNA after chondrogenic differentiation for 10 days. Since sequentially passaged chondrocytes exhibited decreases in SZP expression [11], it is noteworthy that MDMSCs could maintain SZP-secreting phenotype even after passaging.

The present results have important clinical implications. SZP has been considered as a zonal molecular marker for superficial zone of AC. Since the general paradigm of tissue engineering strategies is to mimic the structure of a tissue and native environment as closely as possible to encourage the restoration of its structure and function [12], SZP produced from a superficial layer of tissue-engineered cartilage was considered a critical factor [13]. Gleghorn *et al. *suggested that controlling localization of SZP in engineered cartilage may be critical for proper lubricating function [14]. Quality control, that is, measuring the mechanical properties of the tissue engineered cartilage and in particular of the surface zone, is crucial for generating tissue engineered cartilage constructs that can be used to repair damaged sites in the joints. Notably, the Food and Drug Administration (FDA) recently requested mechanical data for all AC repair products in their guidance for "Repair or Replace Knee Cartilage" (ucm072952) [15], which additionally emphasizes the importance of mechanical characterization of cartilage constructs. Therefore, while quantitative information on the matrix synthesis can be obtained through biochemical analysis and the type and distribution of matrix molecules can be evaluated through immune-labeling, additional measurements, as biomechanical/physical properties, are needed to assess the functional quality of cartilaginous grafts. Fabrication of a functional tissue-engineered cartilage could be dependent on localizing SZP secreting cells at the surface. Our results indicated that MDMSCs may be a useful source of superficial zone chondrocytes with SZP-producing ability. Given its location, accessibility, and great expandability [16], MDMSCs could be a promising cell source for AC tissue engineering.

## Conclusions

The present investigation demonstrated the efficient and reproducible induction of SZP/lubricin accumulation by TGF-β1 and BMP-7 in skeletal MDMSCs. Optimization of the experimental conditions may permit the utility of MDMSCs in generating surface zone-like cells with phenotypic markers of AC and, therefore, constitute a promising cell source for tissue engineering approaches of superficial zone cartilage.

## Abbreviations

7-AAD: 7-Amino-Actinomycin D; AC: articular cartilage; ACI: autologous chondrocyte implantation; ALP: alkaline phosphatase; APC: allophycocyanine; BMP-7: bone morphogenetic protein-seven; FACS: fluorescence-activated cell sorting; FDA: Food and Drug Administration; FITC: fluorescein isothiocyanate; GAPDH: glyceraldehydes 3-phosphate dehydrogenase; HSD: Honestly Significant Differences; MDMSCs: muscle derived mesenchymal stem cells; OA: osteoarthritis; PE: phycoerythrin; PRG: the gene proteoglycan 4; SZP: superficial zone protein; TGF-β1: transforming growth factor-beta one

## Competing interests

All the authors have no financial and personal relationships with other people or organizations that could potentially and inappropriately influence their work and conclusions.

## Authors' contributions

JAA, SCM, PJ-P and AHR participated in the design of the study, acquisition of data, and analysis and interpretation of data, drafting and revising the article for important intellectual content, and gave final approval of the submitted article. SC, JB and TMS participated in the revision of the article and gave final approval of the article for publication. All authors have read and approved the manuscript for publication.
